# Polishing methods for composites restoration: the influence on human gingival fibroblasts behaviour

**DOI:** 10.1186/s12903-024-04418-z

**Published:** 2024-06-03

**Authors:** Benedetta Ghezzi, Matteo Meglioli, Andrea Salvaterra Toffoli, Giovanni Mergoni, Francesca Rossi, Maddalena Manfredi, Simone Lumetti, Edoardo Manfredi

**Affiliations:** 1 Department of Medicine and Surgery, Centre for Dental Medicine, University of Parma, Via Gramsci 14, Parma, 43126 Italy; 2 IMEM-CNR, Institute of Materials for Electronics and Magnetism, National Research Council, Parco Area delle Scienze 37/A, Parma, 43124 Italy

**Keywords:** Dental material, Composite resins, Non-carious cervical lesions, Polishing technique, Oxygen inhibited layer

## Abstract

**Background:**

Carious/Non-carious cervical lesions with gingival recessions may require both dental and periodontal reconstructive therapy, where flaps/grafts may be placed in contact with a dental filling material. Human Gingival Fibroblasts (HGF-1) response during the early phase of healing could vary according to the procedures employed to cure the dental composite. Moreover, oxygen diffusion into dental composite inhibits the polymerization reaction, creating an oxygen-inhibited layer (OIL) that presents residual unreacted monomers. The aim of this study was to assess the effect of different polishing techniques and OIL on HGF-1.

**Methods:**

Composite discs polished with different techniques (diamond rubber, abrasive discs and tungsten carbide burr) were used. An additional not polished smooth group obtained with and without OIL was used as control. Samples were physically characterized through the analysis of their hydrophilicity and surface topography through contact angle measurement and SEM, respectively; afterwards the biologic response of HGF-1 when cultured on the different substrates was analyzed in terms of cytotoxicity and gene expression.

**Results:**

The finishing systems caused alterations to the wettability, even if without a proportional relation towards the results of the proliferation essay, from which emerges a greater proliferation on surfaces polished with one-step diamond rubber and with abrasive discs as well as a direct effect of the glycerin layer, confirming that surface roughness can heavily influence the biological response of HGF-1.

**Conclusions:**

Surfaces wettability as well as cellular behavior seem to be affected by the selection of the finishing system used to lastly shape the restoration. Especially, the presence of OIL act as a negative factor in the regards of human gingival fibroblasts. The present study may provide the first clinical instruction regarding the best polishing system of composite material when the restoration is placed directly in contact with soft tissue cells. Understanding HGF-1 behavior can help identifying the polishing treatment for direct restoration of carious/non-carious cervical lesions associated with gingival recessions.

## Introduction

Carious and non-carious cervical lesions (NCCLs) associated with gingival recessions are common pathological conditions, and their treatment represent a challenge due to both the dental restorative and the periodontal reconstructive aspects, as often NCCLs present a sound margin below the gingival margin, or a deep marginal elevation is needed [1–3]. In fact, when dealing with the cited cases, an optimal polishing of the restoration is decisive in order to allow the proper new attachment of the gingiva and to ensure a long-term success. Indeed, flap stability is a key factor in order to obtain both healing and maturation of the soft tissues [4, 5]. Moreover, the deep margin elevation technique involves placing composite below the gingival level to make the restoration margin more visible and easily isolatable [3]. Therefore, during the polishing, it is important to focus on the creation of the adequate surface where the gingival fibroblasts may find optimal adhesion sites. Dental composites (DC) are the gold standard restorative materials for the treatment of NCCLs, most probably due to their esthetic rendering is easy to be obtained and allow a long lasting surgical procedure [4–6]. Numerous studies already investigated the biocompatibility of DC as chemically complex materials, where the huge variety of monomers and additives influences the biological behavior [7, 8]. The polymerization phase is crucial to avoid the release of non-polymerized monomers through the adjacent tissues or into the oral cavity causing local or even systemic reactions [9, 10]. Obviously the biocompatibility is mainly related to the material itself and to its shrinkage after the polymerization, but some aspects are also linked to dentin permeability and residual thickness of the subject, even if again, the polymerization seems to affect mostly the result [11–13]. Indeed, some in vitro studies underlined how the polymerization reaction is never complete, thus leading to the release of un-polymerized agents not only to the first phase after composite positioning, but also after several days during which residual monomers can spread also systemically [14–16]. Normally, after the initial polymerization, unreacted monomers or additives can spread as degradation factors, as only 55–65% of the whole polymer has properly reacted [17]. The not complete conversion is known to be related to various technical parameters, as the type of curing light, the wavelength, the irradiation time, and the thickness of the filling material’s layer [16, 18, 19]. Nevertheless, it has been seen that also the presence of oxygen can play a pivotal role in the degree of DC polymerization. Oxygen easily reacts with free radicals, leading to the creation of a superficial resin-based layer composed prevalently by unreacted monomers, technically defined as oxygen inhibition layer (OIL) [20–22]. OIL presence leads to a sharp decrease in the degree of initial polymer conversion from 55–80–35% [23–25]. To avoid the permanence of OIL, the final preparation of the DC can be operated by different methods, including the use of diamond rubbers, abrasive discs or tungsten carbide burs, which have been described to produce different finishing microscopical fashions [26]. In the latest years, a change in the way of thinking the design of biomaterials occurred, due to the increased relevance of the close relationship between the cellular components and the extracellular matrix (ECM) [27]. Cell/ECM interactions take place in the micro/nanoscale order, and, since any biomaterial serves as an artificial ECM, its microscopical aspect directs cell behaviour [27]. Numerous studies have been focused on this aspect, highlighting the capacity of cells to specifically respond to distinct spatial nano-patterns and it has been seen that the activation of different signalling pathways (e.g. changes in cytoskeletal conformation, cell shape, motility, proliferation, etc.) is mostly due to differences at the micro-nano level [28–32]. Specifically, at the transmucosal level, gingival fibroblasts have been demonstrated to better adhere on dental implant abutment presenting micro-structured surfaces, if compared to polished counterparts, supporting the idea that the surface processing technique and manufacturing have a great impact on the soft tissue cells response [33–35]. Considering these premises, it is reasonable to hypothesize that the polishing methods used for the refinement of restorations may create peculiar nano-micro profiles, which induce a differential biological response of gingival fibroblasts during their adhesion. Hence, the aim of this study has been to evaluate how the polishing technique used to finish DC or the presence of OIL may influence surface characteristics, such as micro-topography and wettability, and thus the cellular response of gingival fibroblasts.

## Materials and methods

Composite discs polished with four different techniques have been observed in terms of surface topography, wettability and cellular response, in order to determine the optimal finishing method for restorative purpose.

### Sample discs preparation

A hybrid composite of urethane dimethacrylate (UDMA) and other dimethacrylates-based co-monomers (G-Aenial Anterior A2, GC, Italy), whose composition is detailed in Table 1, was shaped to create composite discs. Samples were polymerized with a UV lamp (Dentsply QHL75) with a wavelength of 500 nm for 20 s per side. Subsequently, samples were polished employing three different tools commonly used in clinical practice: one-step diamond rubber (DR - PN20032, Odontoiatrica, Italy), abrasive disc (AD - Optidisc, Kerr Dental, USA) and tungsten carbide burr (TCB - FQC.277.FG.023, Odontoiatrica, Italy). All the samples were polished for 20 s into an aseptic environment and under abundant irrigation with deionized sterile water in order to avoid the creation of artefacts on the surface due to heat development; moreover, each sample was molded, polymerized and polished by the same operator with the same compressive strength, to avoid excessive variability. Smooth surface discs (SM) obtained after compressing the sample with a microscopy slide, was kept with no further treatment, and used as the control group. Moreover, SMG group, was obtained with the same procedure of SM, with the exception that prior the polymerization a thin layer of glycerin (Thermo Fisher Scientific, Carlsbad, CA, USA) was put on the sample, to avoid the formation of the OIL during the polymerization.

**Table 1:** Detail of G-Aenial Anterior A2 (GC) composition

G-Aenial Anterior Composition	Concentration
Urethanedimethacrylate	15–20%
Dimethacrylate	5–10%
Silicon dioxide	25–30%
Composite filler (Pre-polymerized Strontium, Lanthanoid Fluoride and Silica containing fillers − 16–17 μm, Inorganic Silica fillers - >100 nm and Inorganic Fumed Silica filler - <100 nm)	45–50%
Pigments	Traces
Catalysts	Traces

### Surface characterization

In order to analyze the level of surface wettability and the micro-topography of the surface, water-in-air contact angle (CA) analysis and scanning electron microscopy (SEM) investigation were performed. For the measurement of the CA (Ɵ), a 10 µl water drop was settled on samples surface and high-definition images were taken through a camera (Canon EOS 80D) with a macro lens (Canon EF 100 mm 2,8 L) and a ring flash (Canon Macro Twin-Lite MR-14 EX II), using standardized position, exposure and lighting. CA was measured through the ImageJ software (NIH, Bethesda, MA; USA). Samples micro-topography was analyzed through SEM imaging with a dual beam Zeiss Auriga Compact system (Carl Zeiss, Oberkochen, Germany) equipped with a GEMINI Field-Effect SEM column, at 5 keV. Semi-quantitative analysis of the obtained images was performed with the Nikon BR 5.11 Software (Nikon, Tokyo, Japan).

###  Biological behavior

#### Cell culture

Human gingival fibroblasts (HGF-1) were obtained from the American Type Culture Collection (LGC Standards S.r.L., Sesto S.Giovanni, MI, Italy) and cultured in D-MEM high glucose (D-MEM high glucose 4,5 g/l, Thermo Fisher Scientific, Carlsbad, CA; USA) supplemented with 10% Fetal Bovine Serum (FBS, Thermo Fisher Scientific), 1% L-Glutamine (Thermo Fisher Scientific) and 1% Penicillin and Streptomycin (PenStrep, Sigma Aldrich, St. Louis, MI; USA). Cells were kept in an incubator at 37 °C with 5% ppCO_2_ and humidified atmosphere during the whole experimental time.

#### Indirect contact cytotoxicity assay

To exclude the release of any cytotoxic agents from the DC after the polishing treatments, an indirect contact cytotoxicity test was performed in accordance with ISO 10993-5 guidelines for the cytotoxicity analysis of porous materials. To this purpose, the discs were submerged with complete medium (1 mg of material each ml of fresh medium) and stored at 37 °C and 5% CO_2_ in humidified atmosphere for 1 and 10 days. At experimental time points, extract medium was collected and diluted with pristine medium at percentages of 0%, 50%, 70% and 100% of the total final volume. Obtained medium was then added to previously seeded HGF-1 for 24 h. Finally, a CellTiter-Glo (Promega, Madison, WI, USA) chemiluminescent assay was performed to provide a quantification of the number of viable cells in culture by quantitating the amount of ATP present, which indicates the presence of metabolically active cells as a direct relationship between ATP production and cellular viability. Briefly, depleted medium was discarded, samples were rinsed in PBS and a 50:50 solution of DMEM: CellTiter-GLO Lysis Buffer was added. Each sample was incubated 2 min on an orbital shaker, the solution was then collected, luminescence was stabilized for 10 min in the dark and luminescence was measured with a luminometer with double injectors (GLOMAX 20/20, Promega). All the obtained results were normalized to the positive control of cells cultured onto plastic wells which value was assumed as 100% of viability. Final luminescence was then measured with a luminometer with double injectors (GLOMAX 20/20, Promega).

#### Cell proliferation

For the analysis of cell proliferation, sample discs were settled in 48-multiwell plates and HGF-1 cells were seeded at a final density of 15 × 10^3^ cells/well and cultured up to 14 days. Culturing medium was replaced every 3 days. At each experimental time point (1, 3, 7 and 14 days), the proliferation was assessed by chemiluminescence (CellTiter-GLO, Promega Corporation, Madison, WI; USA) assay, as described above.

#### Gene expression analysis

After 10 days from HGF-1 seeding onto DC discs, their differentiation into mature synthetizing ECM cells was studied. Briefly, total RNA was extracted through a RNeasy® Mini Kit protocol (Qiagen, Hilden, Germany), and used as a template for cDNA synthesis (High-Capacity cDNA Reverse Transcription Kit, Applied Biosystems, Forster City, CA, USA). Quantitative RT-PCR experiments were then performed for the relative expression of Caspase 3, Cyclin Dependent Kinase 2 (CDK2), Transforming Growth Factor Beta (TGF-β) and Interleukin 6 (IL-6) was investigated. Glyceraldehyde-3-phosphate dehydrogenase was used as housekeeping gene.

### Statistical analysis

Values of each group have been reported as means ± standard deviation (SD) of three independent experiments performed with four technical replicates each. Statistical analysis was performed using Prism 8 (GraphPad, La Jolla, CA, USA). After the initial validation of the normal distribution of the data through Shapiro-Wilk test, the differences among groups was assessed by a ONE-way ANOVA, TWO-way ANOVA for repeated measures and Tukey’s post hoc test to identify significant differences. Differences were considered significant when *p* < 0,05.

## Results

### Surface characterization

**Fig. 1:**
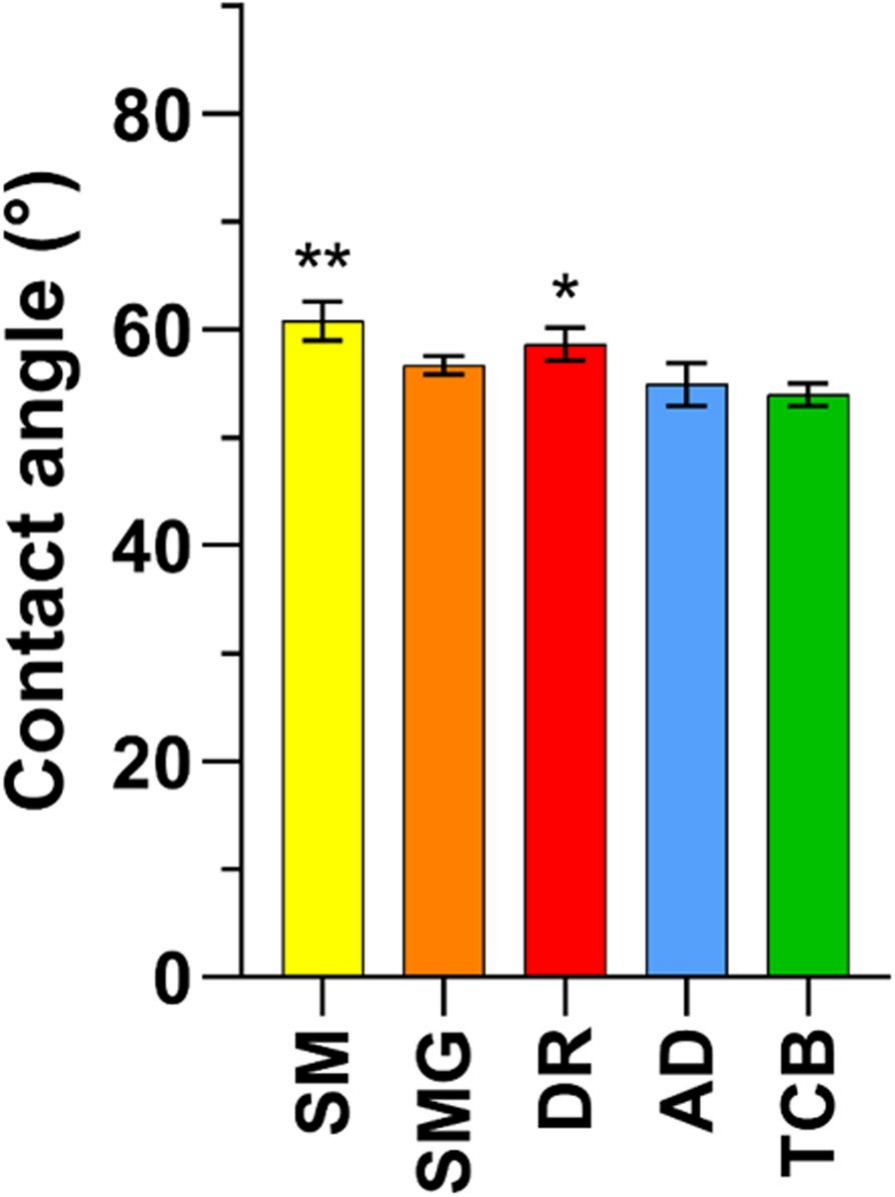
Bar chart of the contact angle measurement of every experimental group with the summary of the statistically significant differences among the groups. *p* = 0,0007 SM vs. SMG; *p* < 0,0001 SM vs. AD and SM vs. TCB; *p* < 0,0001 DR vs. TCB and *p* = 0,0019 DR vs. AD

**Fig. 2:**
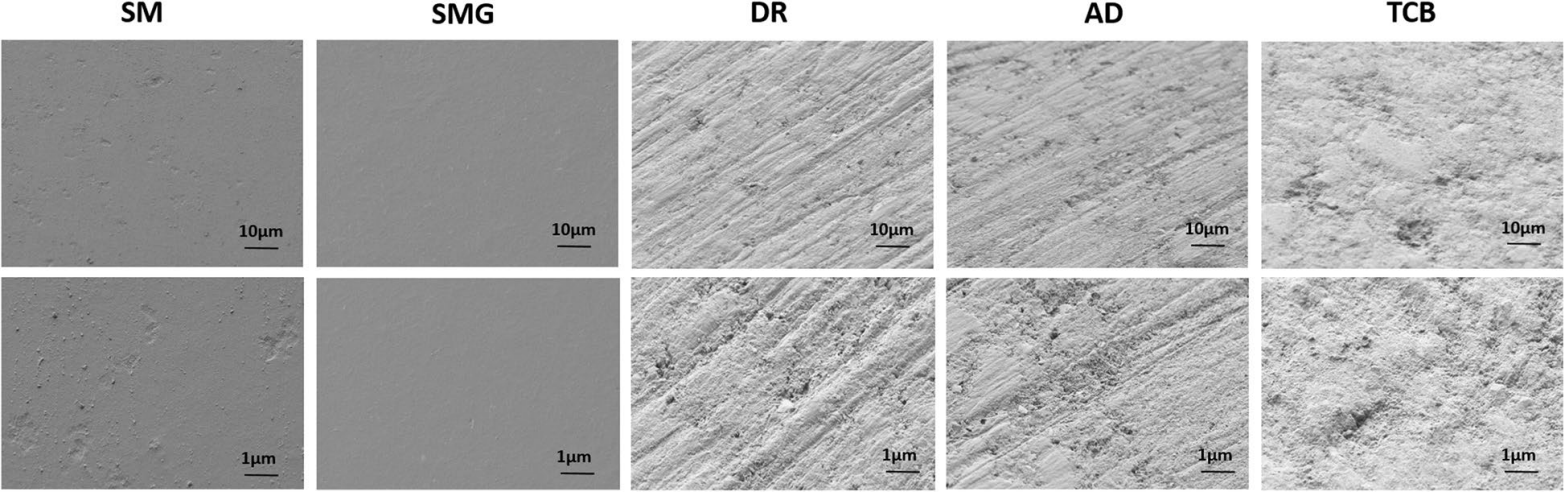
Scanning Electron Microscopy images of the composite resins samples at smaller (upper line) and bigger (lower line) magnifications

**Fig. 3:**
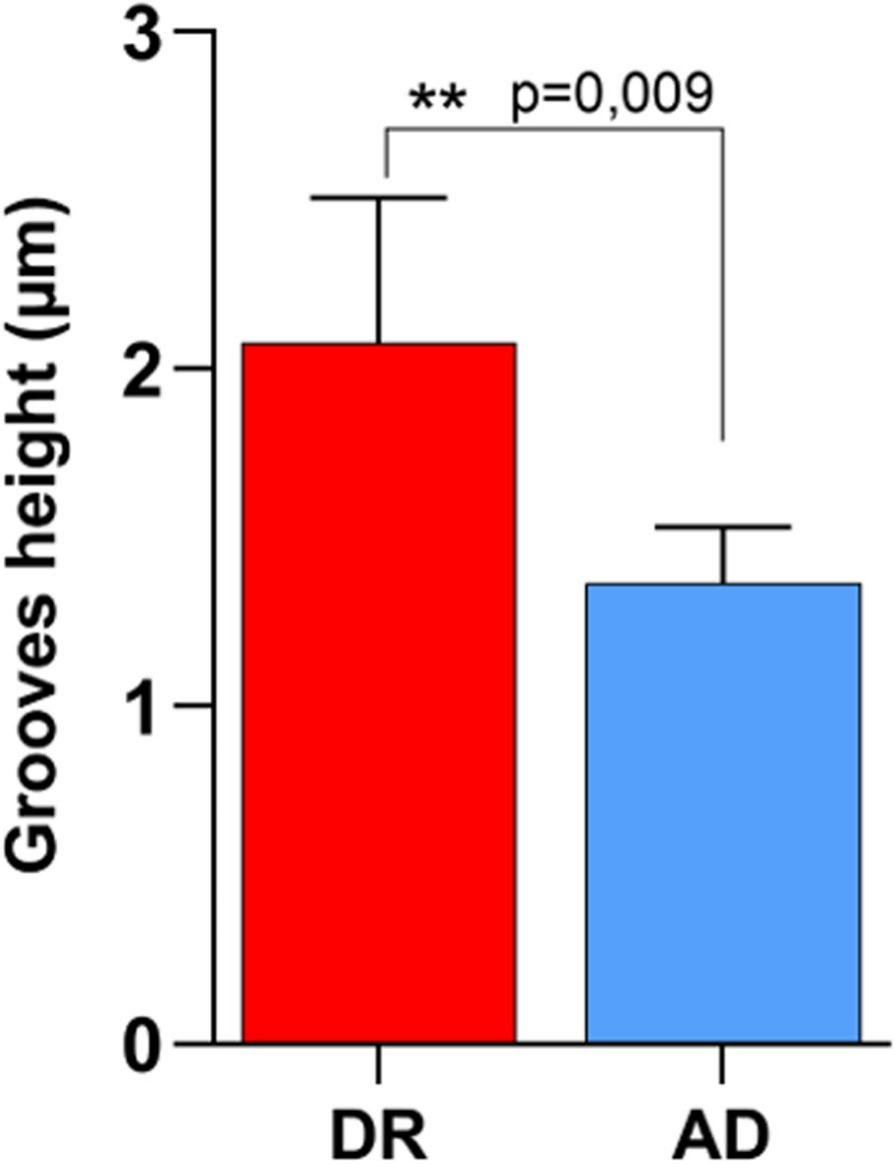
Graph showing the detail of grooves depth measurement on DR and AD samples. **p = 0,009

The analysis of surface hydrophilicity was carried out through the water-in-air contact angle measurement (Fig. 1). AD and TCB samples showed a significant increase of the surface wettability if compared to the SM control group, as well as it occurred for the SMG samples. However, no change of surface wettability was recorded for the DR group, which hydrophilicity was comparable to that of the control and significant diverged from that of the AD and TCB group. Major differences among the composite polishing techniques occurred at the micro-topographical level, as shown in SEM microphotographs (Fig. 2). At small magnification (upper line) the control groups (SM and SMG) appear smooth and regular, while the discs polished with DR and with AD clearly present holes and stripes; lastly, TCB samples showed a completely rough surface with no specific patterns to be detected. Noticeably, DR samples presents horizontals and parallels stripes, while the AD samples possess less regular and slightly curved ones. The analysis of the grooves depth shown in Fig. 3 was carried out starting from the highest magnification images (lower line, Fig. 2) and underlined that there was a statistically significant difference between the height of AD and DR samples (*p* = 0,009). Additionally, as it is obvious that in all the polished samples, filler particles and porosities are evident on the surfaces as small holes into the resin composite, with particular regard to the TCB samples.

###  Gingival fibroblasts response

HGF-1 behavior on DC finished with different polishing tools and techniques has been quantified to clarify the role of surface micro-topography and wettability on cellular responses.

**Fig. 4:**
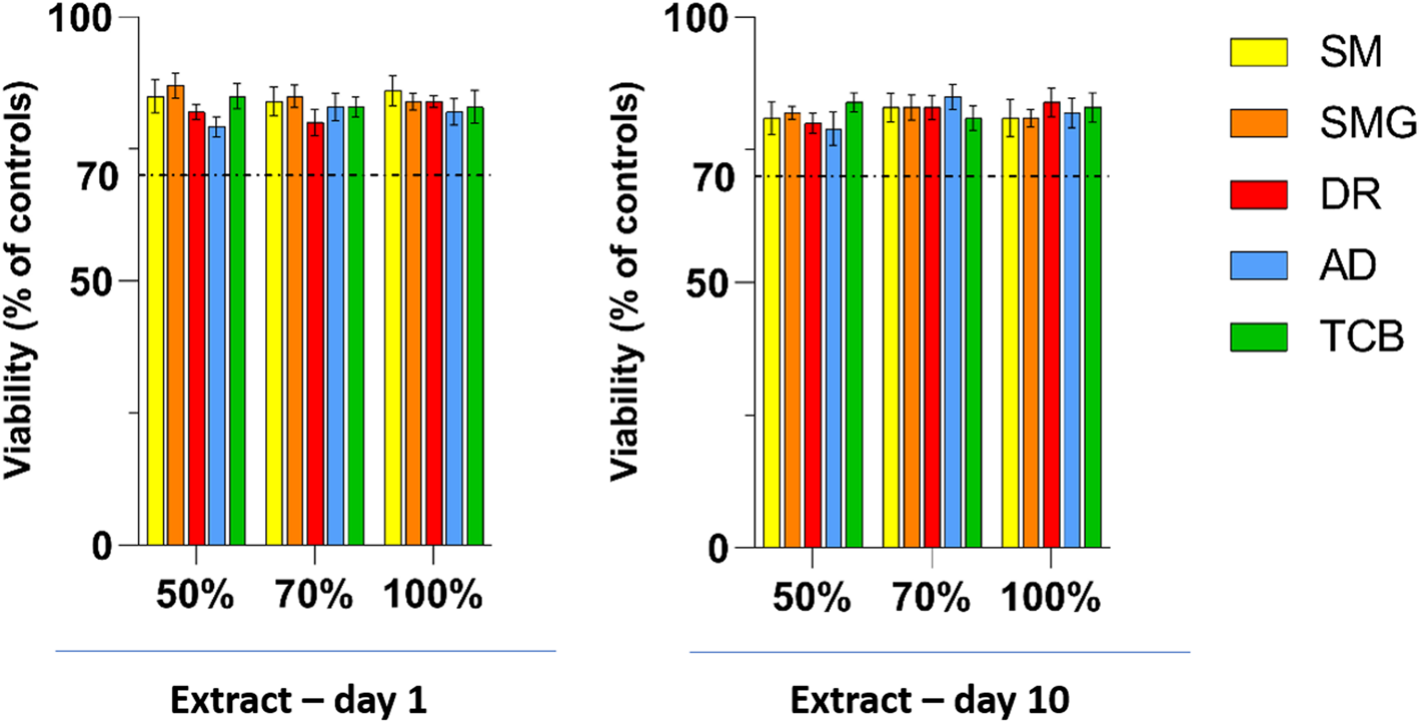
Indirect cytotoxicity assay performed after 1 and 10 days after discs immersion in the culturing medium. All the samples exceed the 70% limit of the control samples to consider the biomaterial as not cytotoxic

**Fig. 5:**
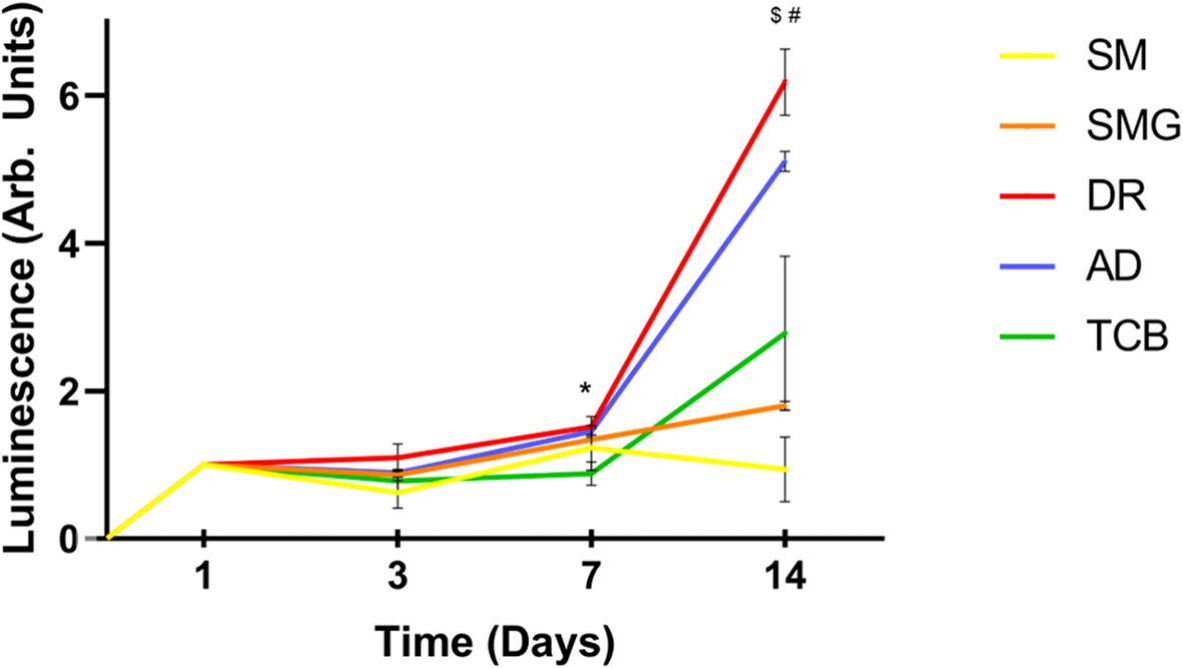
Analysis of the mean values of luminescence (normalized to t1) of each group at every experimental time point. *p < 0.05 AD and DR vs. TCB; $ *p* < 0.001 SMG vs. SM; # *p* < 0.001 AD, DR, TCB vs. SM

Figure 4 shows the viability of fibroblasts after being cultured for 24 h in the previously extracted medium and all the experimental groups had a viability of over 70% at both the time points, as requested from ISO Guidelines in the evaluation of the cytotoxicity of porous materials. Moreover, the results shown in Fig. 5 underlined a first significant difference only seven days after the seeding: both groups, DR and AD, showed a greater proliferation if compared to the TCB group. Interestingly, at the last experimental time point, these differences arise increased and all the post-cured group appeared statistically significant if compared to the control SM group. Noteworthy, even if all the polished samples showed a higher proliferation if compared to the SMG samples, also the SMG group expressed an increased proliferation rate when compared to the samples without glycerin. Nevertheless, DR group had the highest increase in cell number, strictly followed by AD samples.

### Gene expression analysis

Gene expression analysis on HGF-1 cells cultured onto the alternatively polished substrates has been performed to evaluate the possible impact of the different polishing in the regards of human fibroblasts. In particular, two genes linked to cellular health have been investigated focusing on the control of the execution-phase of cell apoptosis (Caspase 3), the progression through cell cycle (CDK2). In parallel, two genes related to the inflammatory and anti-inflammatory pathways have been studied to understand if there is a possible link among the different treatments and chemokine release.

**Fig. 6:**
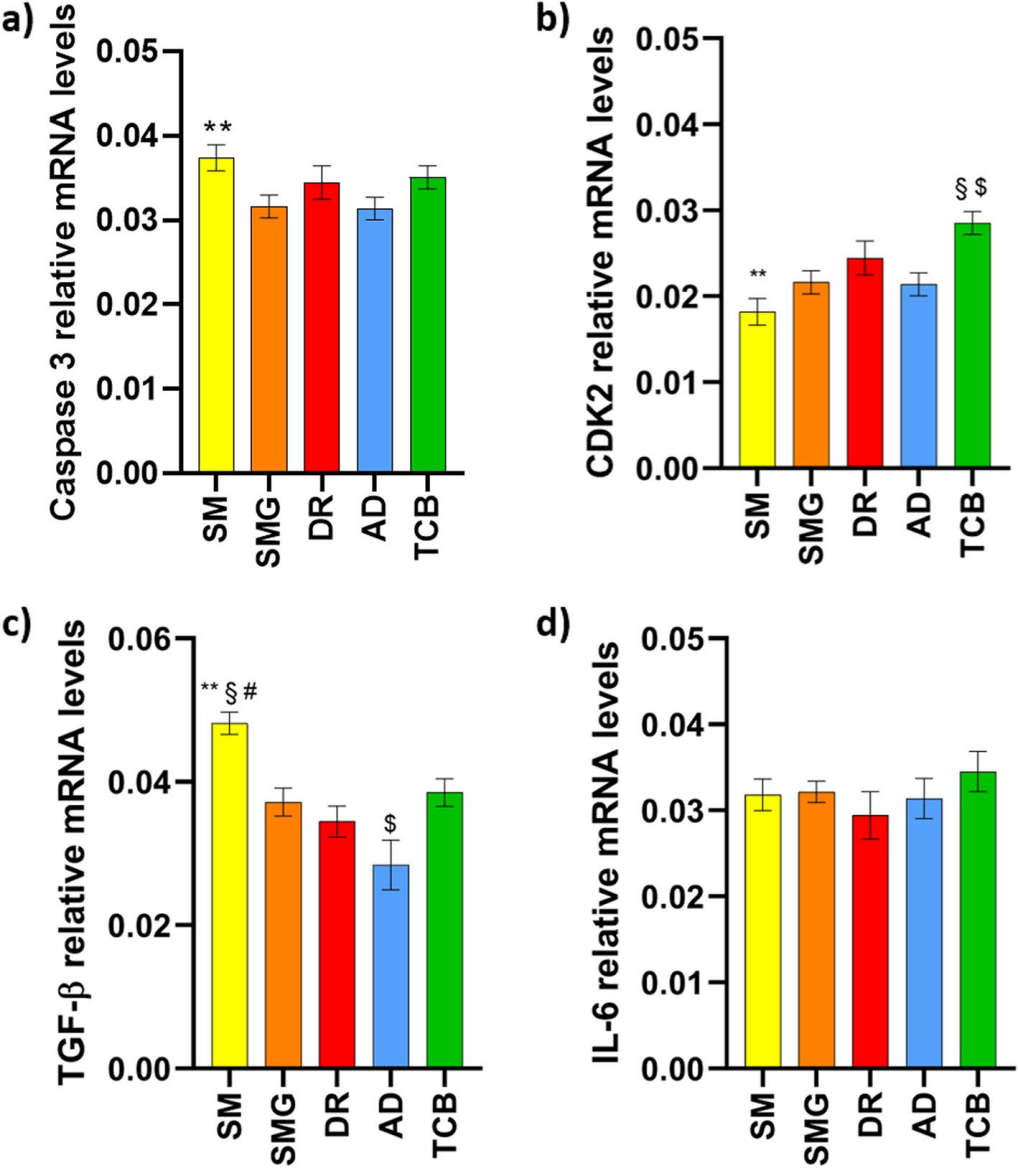
RT-PCR analysis of the gene expression of (**a**) Caspase 3, **p < 0.05 SM vs. SMG and SM vs. AD; **b** Cyclin Dependent Kinase 2 (CDK2), **p < 0.05 SM vs. DR, $ *p* < 0.0001 TCB vs. SM, § *p* < 0.05 TCB vs. AD and TCB vs. SMG; **c** Transforming Growth Factor Beta (TGF-β), **p < 0.05 SM vs. SMG and SM vs. TCB, § *p* < 0.01 SM vs. DR, # *p* < 0.0001 SM vs. AD, $ *p* < 0.05 AD vs. SMG and AD vs. TCB and **d** Interleukin 6 (IL-6)

The analysis of mRNA (Fig. 6) showed a Caspase 3 expression in SMG and AD groups significantly lower if compared to the SM group; there were no differences to report in the IL-6 expression, indicative of a not activated inflammatory response. Nonetheless, both CDK2 and TGF-β were differentially expressed among the groups. CDK2, whose activity is especially critical during the G1 to S phase transition showed the higher expression in TCB samples (**p < 0.05 SM vs. TCB), and the absolutely lower in the control SM (*p* < 0.05 AD vs. SMG and AD vs. TCB), indicating a higher proliferation activity of HGF-1; on the other side, SM expression of TGF-β was the highest, underlining an alteration in gene expression most likely due to the presence of OIL. DR and AD groups showed the lower level of TGF-β, while a slight increase has been detected in SMG.

## Discussion

The present study addressed the apparent faint biologic rationale at the cellular level underlying a common clinical procedure of restorative dentistry. Restorative therapies sometimes require a strong and long lasting attachment of the gingival tissue upon the composite material. The use of an optimal polishing technique can favor both soft tissue attachment and healing, as well as the creation of an adequate root profile where the gingival fibroblasts may find optimal adhesion sites. Furthermore, in those cases where the use of polish is not required, OIL may cause a number of problems related to the presence of uncured resin on the surface of the biomaterial and the consequent release of monomers [36]. Due to the importance of the polymerization reaction not only on the biological effectiveness of the material, but also for its hardness, as well as for aesthetic discoloration of DC, it is mandatory, especially in clinical practice, to optimize the polymerization reaction conditions [20]. The goal of this study was to define the most “fibroblast-friendly” polishing method for composite resins in an in vitro cellular model and, in parallel, to verify if the use of a glycerin layer during the polymerization has an impact in the regards of the biological response to OIL. Obtained data highlighted the importance of the polishing technique in the development of surface micro-topography, confirming the literature data indicating that each different finishing method affects cellular behavior [28]. As observed through SEM imaging, the SM samples presented a smooth surface, in contrast with all the polished samples. Especially DR and AD surfaces presented holes and stripes which can be compared to the typical surface aspect of machined titanium implants, while the TCB samples showed a more homogeneously distributed roughness, typical of sandblasted-acid etched implants [35]. As it is already been described for trans-gingival titanium surfaces, surface roughness plays a pivotal role in many biological processes, as protein adsorption or soft tissues cellular adhesion and migration, which has been also confirmed by the proliferation assay on DC resin [37]. The analysis of the contact angle showed that AD and TCB polishing significant increased the hydrophilicity of the material, while on the other hand, no significant increase was recorded when DR was used. Noteworthy, there is a statistically significant difference in the hydrophilicity between SM and SMG groups, probably because the presence of a glycerin layer during the polymerization of the SMG samples has allowed the complete polymerization of the external layer, avoiding the formation of a sticky resin interface that could have limited the spreading of the liquid, consequently improving the homogeneity of the samples [38]. Several in vitro studies have investigated HGF-1 proliferation over materials with different wettability, with predominant interest to titanium surfaces, with the intent of addressing the trans mucosal biological and clinical aspect of implant-supported prosthodontics; regretfully, not many studies investigated this aspect on DC after different polishing, thus making it difficult to obtain a direct comparison with other findings [39, 40]. Our data did not underline a statistical difference among the samples at the first time point. Nevertheless, moving to the results of cell proliferation and correlating them to contact angles and the morphological effect of polish, but up to 7 days, differences were found among DR and AD groups in the regards of TCB polishing. Pursuing the 14 days of testing all the experimental groups showed statistically increased cell proliferation if compared to the SM control, showing, consistently with the literature, that both topography and hydrophilicity are key factors to be considered to favor cell response [35, 41, 42]. It does leaps to the eye that there is a delayed response of HGF-1 cells effect that could be related to the replication rate of this specific cellular line. Lastly, also SMG samples showed a higher cell proliferation than SM, corroborating the idea that the lack of OIL can favor cell adhesion adhere and proliferation. The obtained results showed the positive effect of DR, a surface that morphologically remember a machined trans-gingival titanium surface, on fibroblastic response in terms of cellular proliferation, showing the positive effect of a “simil-machined” surface on fibroblasts behavior [43, 44].

Unfortunately, to the best of our knowledge, it is not possible to directly compare the result with other studies, as the most of them included 3T3 fibroblasts, thus creating an environmental setup that cannot mimic the in vivo condition. Moreover, in some studies where HGF-1 were used, the experimental protocol is limited to shorter time points or is not related to different polishing techniques, but in the most of the cases to different resins, not taking into consideration the effect of the final treatment [13, 22, 24, 45–48]. Furthermore, SM samples are the only ones that presented a decline in cellular proliferation within the seventh and the fourteenth day; this is evident and even if not statistically significant, it worth a closer look. This fact might indicate cellular suffering, which, however, was not visible on the SMG group and that might be related to an inhibited polymerization of the external layer by OIL [49]. This observation could be the key to assess that during restorative procedures with composite materials, the polymerization of the external layer has to occur with the presence of a glycerin gel, to avoid the contact between the dental resin and the atmospheric oxygen. Many research outcomes have demonstrated how micro-topography and geometric cues of the substrates affect cellular adhesion and proliferation [50–53]. In particular Chen et al. demonstrated that fibroblasts NIH/3T3 show a higher adhesion on a glass surface with a micro-rough pattern than on a smooth one, supporting what has been observed in the present study [54]. Lastly, we aimed to understand if the polishing technique could also influence the differential gene expression of HGF-1 cells. As shown in Fig. 6, the level of transcripts related to cell cycle and cell death are lower and higher, respectively, in the SM group; the level of TGF-β on SM was much higher than in all the other experimental groups, while no statistical significant results were found on the IL-6 side. The incidence of apoptotic markers as Caspase 3 supports the idea that some topographies (especially SM) might be related to an increase in the inflammatory response, as also corroborated by the increase of TGF-β, which acts as controller of the immune response and as anti-inflammatory molecule also playing a pivotal role in the returning to the balance in inflammatory periodontal diseases including gingivitis and periodontitis [55–57]. Indeed, Ilday et al., underlined that the level of some pro-inflammatory interleukins function were found to differ significantly after restorative treatment in vivo, thus suggesting that composite resins monomers might cause some changes or have some negative effects in the oral cavity [58]. We underlined an aspect that has not been investigated before, the effectiveness of specific polishing treatments on a composite material and the behavior of human gingival fibroblasts, thus proposing a new point of view for further in vitro or in vivo studies on the clinical incidence of the selected treatment. In this regards, it could be helpful to deeply analyze the chemical structure of the material, investigating also the conversion degree of the monomers, the molecular composition of the formed OIL or even comparing the effect of the proposed polishing treatments on different dental composite materials. Nevertheless, our study lays the foundation for directing the dentist to specific material processing in order to promote tissue healing and achieve long-term clinical success.

## Conclusion

The obtained results highlighted that all the polishing techniques used in this study determine important modifications to DC surface biological effectiveness, especially through surface wettability and OIL formation. The presence of glycerin during the polymerization increases noticeably the effectiveness of the SM group, decreasing the expression of apoptosis related genes. Our data confirm the non-cytotoxic behavior of G-aenial Anterior DC towards HGF-1 cell line, with peculiar attention to the DR polishing. This study gives the experimental base for further analysis on the relation between HGF-1 and DC topography and to clarify which factors might contribute to its improvement. We in vitro investigated a fundamental issue of daily practice, which has not been properly explored in literature so far, thus this study may provide the first clinical suggestion regarding the best polishing system of DC when it has to be placed directly in contact with soft tissue cells.

## Data Availability

The data that support the findings of this study are available from the corresponding author upon reasonable request.
